# Influenza Vaccination Status and Associated Factors Among Older Adults in Suzhou, China: A Comparative Cross-Sectional Survey

**DOI:** 10.3390/vaccines14070645

**Published:** 2026-07-22

**Authors:** Ningning Du, Shuai Shao, Yuanyuan Zhang, Cheng Liu, Yuanyuan Pang, Hui Hang, Liling Chen

**Affiliations:** 1 School of Public Health, Nanjing Medical University, Nanjing 211166, China; duningning@stu.njmu.edu.cn (N.D.); shaoshuai@stu.njmu.edu.cn (S.S.); 2 Suzhou Center for Disease Control and Prevention, Suzhou 215131, China; zhangyuanyuan@szcdc.cn (Y.Z.); liucheng@szcdc.cn (C.L.); pangyuanyuan@szcdc.cn (Y.P.); hanghui@szcdc.cn (H.H.)

**Keywords:** older adults, influenza vaccine, vaccination uptake, associated factors, knowledge, attitudes, practices (KAP)

## Abstract

**Background:** Influenza poses a serious threat to the health of older adults, and vaccination is a key strategy for prevention. As an economically developed city, Suzhou has a rapidly aging population, yet the influenza vaccination rate among older adults remains substantially lower than that in developed countries. There is an urgent need to understand the current vaccination status and its associated factors to inform future strategies for improving vaccination coverage. **Objectives:** To compare influenza- and vaccine-related knowledge, attitudes, practices, and information access channels between vaccinated and unvaccinated older adults (≥60 years) in Suzhou, and to identify factors associated with vaccination behavior in economically developed areas. **Methods:** A comparative cross-sectional survey was conducted from April to August 2024 across six districts or county-level cities randomly selected from Suzhou’s ten county-level administrative divisions. Participants were divided into vaccinated and unvaccinated groups based on their influenza vaccination status during the 2023–2024 influenza season. A two-stage sampling method was employed: the six districts were selected as primary sampling units; within each district, eligible older adults (aged ≥60 years) were randomly selected from two independent sampling frames—the vaccination information system for the vaccinated group and the basic public health service system for the unvaccinated group. Face-to-face structured interviews were conducted using a questionnaire consisting of four sections; data were entered using EpiData 13.1 and analyzed using SPSS 27.0. The χ^2^ test, *t*-test, and multivariable logistic regression were used for statistical analysis. **Results:** Among 4622 valid older adults (vaccinated: *n* = 2007; unvaccinated: *n* = 2615), the vaccinated group scored significantly higher on influenza-related knowledge (4.06 ± 2.21 vs. 3.16 ± 2.33, *p* < 0.001), vaccine-related knowledge (2.10 ± 1.18 vs. 0.67 ± 1.07, *p* < 0.001), and perceived influenza risk scores (2.72 ± 1.42 vs. 2.26 ± 1.64, *p* < 0.001). Vaccine safety and efficacy endorsement were also markedly higher among the vaccinated group (70.3% vs. 22.0%; 49.7% vs. 9.3%). Regarding information sources, the vaccinated group relied more on healthcare institutions (54.56% vs. 46.92%), whereas the unvaccinated group relied more on television (60.34% vs. 51.17%). Multivariable logistic regression revealed that awareness of the influenza vaccine (aOR = 9.151, 95%CI: 6.32–13.25), having a planned vaccination site (aOR = 2.66, 95%CI: 2.01–3.54), and perceiving the vaccine as safe (aOR = 1.81, 95%CI: 1.31–2.49) were positively associated with vaccination, while rural household registration (aOR = 0.76, 95%CI: 0.63–0.93), full-time employment (aOR = 0.41, 95%CI: 0.28–0.60), and hypertension (aOR = 0.79, 95%CI: 0.65–0.94) were inversely associated. **Conclusions:** Compared with the unvaccinated group, the vaccinated group demonstrated significantly better influenza- and vaccine-related knowledge, risk perception, and recognition of vaccine safety and efficacy, and relied more on healthcare institutions for information. Multivariable regression analysis identified awareness of the influenza vaccine as the strongest associated factor. These findings suggest that vaccination coverage could be improved by strengthening vaccine knowledge promotion through healthcare professionals and increasing awareness of vaccination policies that integrate medical insurance reimbursement.

## 1. Introduction

Influenza causes approximately 3 to 5 million severe cases and 650,000 respiratory deaths worldwide each year, posing a serious threat to public health [1]. Older adults often experience a decline in immune function due to immune senescence, and their risk of developing severe illness, hospitalization and death after being infected with influenza is significantly higher than that of healthy adults. A sampling survey conducted in eight cities in China from 2003 to 2008 showed that 86% of influenza-related deaths occurred among people over 65 years old [2].

Elderly people are often accompanied by one or more chronic underlying diseases such as hypertension, diabetes, and chronic obstructive pulmonary disease, and their abnormal levels of immune factors such as immunoglobulins further reduce their immunity to influenza [3]. A number of studies have shown that concurrent infectious diseases often aggravate the original chronic diseases, which is one of the important reasons leading to the increased mortality of people with chronic diseases [4,5,6]. Influenza vaccination is the most cost-effective way to prevent influenza and its complications, which can reduce the risk of influenza morbidity and mortality. A number of studies have confirmed that influenza vaccine can reduce the mortality of people with chronic diseases [7,8], which is of great significance for reducing influenza-related complications in patients with chronic diseases [9]. Both the World Health Organization and Chinese Center for Disease Control and Prevention recommend older adults as the priority population for influenza vaccination. The median influenza vaccination rate of older adults in developed countries such as Europe and the United States has reached 45.7% [10], while the average vaccination rate of older adults over 60 years old in China is only 3.8% [11].

Suzhou is an economically developed area in China. The population of older adults aged 60 and above has reached 1.87 million in the permanent population, but the influenza vaccination rate of older adults is very low. The influenza vaccination rate is less than 1% [12], which is significantly lower than the national average and even lower than that of developed countries. In Suzhou, influenza vaccine is classified as a category II (non-National Immunization Program vaccine) vaccine and administered on a voluntary and self-financed basis. The optimal timing for vaccination is early before the influenza season, ideally by the end of October. Several formulations are available, including trivalent inactivated, trivalent live-attenuated, and quadrivalent inactivated vaccines, with either trivalent or quadrivalent types recommended for older adults. Influenza vaccination services are available at community health service centers and designated vaccination clinics across all districts. Regarding medical insurance reimbursement policy, the Jiangsu Provincial Medical Security Bureau announced that, effective 1 January 2023, medical insurance personal accounts may be used to cover the costs of non-immunization program vaccines, including influenza vaccines. Additionally, during the 2025–2026 season, some districts implemented temporary subsidy initiatives: Suzhou Industrial Park introduced a 50% subsidy for quadrivalent influenza vaccines, and Gusu District provided free 23-valent pneumococcal polysaccharide vaccination for residents aged 65–84 years, reflecting ongoing efforts to enhance vaccine accessibility for older adults.

Therefore, a systematic evaluation of knowledge, attitude and practice (KAP) about influenza and influenza vaccine among older adults in Suzhou can provide a scientific basis for formulating targeted intervention measures and improving vaccine coverage in economically developed areas. This study aims to evaluate the status of influenza vaccination in older adults in Suzhou, analyze the differences between the vaccinated group and the unvaccinated group on influenza- and influenza vaccination-related knowledge, attitude and practice (KAP), and identify the key factors associated with vaccination status, so as to provide reference for the optimization of influenza vaccination policy.

## 2. Materials and Methods

### 2.1. Subjects

The survey was conducted from April to August 2024, following the conclusion of the 2023–2024 seasonal influenza epidemic period. A two-stage sampling method was employed: first, six districts or county-level cities were randomly selected from the ten county-level administrative divisions of Suzhou; second, within each selected district/county, eligible older adults (aged ≥60 years, permanent residents) were randomly drawn from two independent sampling frames based on their influenza vaccination status during the past year—the vaccination information system for the vaccinated group and the basic public health information system for the unvaccinated group. Participants who were not registered in the corresponding information system or were non-local permanent residents were excluded.

According to the Suzhou Bureau of Statistics, the total number of permanent residents aged ≥60 years in Suzhou was 3.26 million. Across the six selected districts, the basic public health information system covers approximately 80% of the local older adult population, providing the sampling frame for the unvaccinated group, while the vaccination information system served as the frame for the vaccinated group. A total of 5000 older adults were contacted, yielding 4622 valid questionnaires (2007 vaccinated and 2615 unvaccinated), with an overall effective response rate of 92.4% (91.2% for the vaccinated group and 93.4% for the unvaccinated group).

This survey has been approved by the Ethics Committee of the Suzhou Center for Disease Control and Prevention (approval number: SZCDC/IRB/PJ/2024-06). All the subjects voluntarily participated in the survey and signed an informed consent.

### 2.2. Sample Size Calculation

Sample size was estimated based on a formula for comparing rates in two independent groups:

$$n=\frac{{\left(Z_{\alpha /2}\sqrt{2p_{c}(1-p_{c})}+Z_{\beta }\sqrt{p_{1}(1-p_{1})+p_{2}(1-p_{2})}\right)}^{2}}{{\left(p_{1}-p_{2}\right)}^{2}}$$

Here, $p_{c}={(p}_{1}+p_{2})/2$.

Based on the results of our pilot survey data, we assumed the following parameters: p1 (expected knowledge rate in the vaccinated group) = 65%; p2 (expected rate in the unvaccinated group) = 50%. With a two-sided significance level of α = 0.05 (Zα/2 = 1.96), power of 80% (Zβ = 0.84), and a 1:1.5 allocation ratio (vaccinated: unvaccinated), the required sample size was calculated as 1560 vaccinated and 2340 unvaccinated (total 3900). Considering the 20% non-response or invalid questionnaire rate, the final planned sample size was 5000 people. In the actual implementation, the allocation ratio was adjusted to approximately 1:1.3; the final sample size of 4622 (2007 vaccinated and 2615 unvaccinated) remained well above the minimum requirement of 3900.

### 2.3. Survey Methods and Contents

This study used a uniformly designed questionnaire, which was revised by several rounds of expert review and a pilot survey to ensure the reliability and validity of the questionnaire. Cronbach’s α coefficients for the knowledge sections were 0.841 and 0.835, and EFA confirmed a two-factor structure (KMO = 0.905, Bartlett’s test *p* < 0.001). The complete questionnaire is provided in Supplementary S1. The survey covered basic information, life history, family economic status, health status and lifestyle, awareness of influenza and influenza vaccination, influenza vaccination intention, access to knowledge about influenza prevention and control, and exploration of factors associated with influenza vaccination willingness.

Sociodemographic and health-related variables included sex (female and male), age (continuous), household type (urban and rural), medical insurance type (no insurance, Resident Basic Medical Insurance, Employee Basic Medical Insurance, New Rural Cooperative Medical Scheme, and other commercial insurance), current employment status (retired, part-time, or full-time), presence of students or children in the family, personal annual income, smoking status, self-rated health status, and chronic conditions (diabetes, hypertension, cancer, and so on).

Knowledge variables included influenza-related knowledge score, vaccine-related knowledge score, awareness of influenza (yes/no), level of influenza knowledge, awareness of influenza vaccine (yes/no), and level of vaccine knowledge. Knowledge and risk perception were assessed using scoring methods, with higher scores indicating better knowledge or greater perceived risk. Attitudinal and behavioral readiness variables included vaccine safety, vaccine effectiveness, influenza risk perception score, behavioral readiness, vaccination safety concerns, accessibility to services, subjective norms, perceived protection, and perceived benefits of vaccination.

All the investigators received unified training and assessment. After obtaining the informed consent of the respondents, a face-to-face interview was conducted to complete the paper questionnaire survey.

### 2.4. Quality Control

The investigators checked and cleaned the questionnaires every day and checked for missing items. A double-entry method was used to input the paper questionnaire data into the questionnaire star platform to form an electronic database. Influenza vaccination information was verified through the Suzhou adult vaccination platform to ensure accuracy. The centers for disease control and prevention of each jurisdiction were responsible for on-site technical guidance and data quality control, and documented supervision feedback and corrective actions. The project team of the Suzhou Center for Disease Control and Prevention conducted on-site supervision and random checks of the questionnaires to ensure the authenticity and validity of the data.

### 2.5. Assignment Methods

Influenza- and influenza vaccine-related knowledge were evaluated using a scoring method, and each question was worth 1 point. Incorrect answers or “do not know” were not scored. If a single choice question is correct, 1 point is awarded. For the multiple-choice question, n correct choices are given (1/n) points, and the total score is calculated. Of the 10 influenza knowledge questions, 8 were scored (covering severity, transmission routes, prevention measures, and epidemic season). Two questions were excluded from scoring: (1) “awareness of influenza” (yes/no), a binary variable; and (2) “level of influenza knowledge” (nothing/a little/some/a lot/a great deal), an ordinal categorical variable. Similarly, of the 7 influenza vaccine knowledge questions, 5 were scored (optimal timing, locations, frequency, priority populations, and reimbursement policy). The two items on “awareness of influenza vaccine” (binary) and “level of vaccine knowledge” (ordinal) were likewise excluded from scoring. The section on influenza-related knowledge was scored out of 8 points, and the section on influenza vaccine was scored out of 5 points, for a total of 13 points.

### 2.6. Statistical Analysis

All data were entered by EpiData 13.1 software. Excel was used to establish a survey database of influenza knowledge, attitude and behavior of older adults in Suzhou. After carefully checking the completeness, accuracy and proofreading logic of the data, SPSS27.0 software was used for statistical description and analysis. The measurement data conforming to the normal distribution were expressed as x ± s, the measurement data not conforming to the normal distribution were expressed as M (Q1, Q3), and the count data were expressed as the rate or proportion (%). *χ*^2^ test or Fisher’s exact test was used for univariate analysis, and a binary logistic regression model was used for multivariate analysis. The odds ratio and 95% confidence interval were calculated. The significance level of this study was set as α = 0.05.

## 3. Results

### 3.1. Basic Characteristics of Respondents

A total of 5000 older adults aged 60 years and over were surveyed, and 4622 valid questionnaires were collected, with an effective recovery rate of 92.44%. The average age of the respondents was 70.75 years (70.75 ± 6.93 years). In Table 1, the 60–69 years old, 70–79 years old and ≥80 years old accounted for 47.32%, 41.06% and 11.6%, respectively. 62.5% of them were registered in rural areas; 52.4% of them had primary school education or below. More than half of the population was covered by employee medical insurance (53.4%). The personal annual income was less than 50,000 yuan (69.8%), and the family annual income was between 50,000 yuan and 100,000 yuan (29.6%). Body Mass Index (BMI) in the normal range accounted for 51.6%, 51.6% had hypertension, and 34.1% had diabetes.

### 3.2. Awareness of Influenza- and Influenza Vaccine-Related Knowledge

A total of 10 questions were set up to evaluate the knowledge of influenza in older adults, and seven questions were set up to evaluate the knowledge of influenza vaccine in older adults, covering knowledge of influenza virus, serious adverse outcomes, transmission routes, preventive measures, epidemic season in the Suzhou area, and vaccine awareness, cognitive level, the best time, place, frequency, priority vaccination population, and medical insurance reimbursement policy. The results showed that the awareness rate of both influenza-related knowledge and influenza vaccine in the vaccinated group was significantly higher than that in the unvaccinated group (both *p* < 0.05). It is worth noting that among all the influenza vaccine-related knowledge items, high awareness rates (>40%) were observed for only three: (1) community service centers as vaccination points, (2) individuals aged ≥60 years as a priority group, and (3) reimbursement of vaccination costs by medical insurance. In contrast, the awareness rates for the remaining items were below 40%. The core questions on influenza and influenza vaccine knowledge were further scored separately, with a total score of 8 for influenza knowledge and 5 for influenza vaccine knowledge. See Table 2 for details. The results showed that the average score of influenza knowledge in the vaccinated group was higher than that in the unvaccinated group (4.06 ± 2.21 vs. 3.16 ± 2.33, *p* < 0.05). In terms of influenza vaccine knowledge, the average score of the vaccinated group was higher than that of the control group (2.10 ± 1.18 vs. 0.67 ± 1.07, *p* < 0.05).

### 3.3. Attitudes and Behaviors Related to Influenza and Influenza Vaccination

#### 3.3.1. Influenza-Related Attitudes and Behaviors

In Table 3, four questions were used to evaluate the risk perception of influenza in older adults, including the concern of themselves or their family members suffering from influenza, the risk perception of severe illness, and the susceptibility perception. The results showed that the proportion of the following dimensions in the vaccinated group was significantly higher than that in the unvaccinated group (all *p* < 0.05): worry about influenza in relation to themselves or their families (moderate or higher), self-rated risk of severe disease, and self-rated susceptibility compared with peers. Further scoring the four questions of risk perception (a total score of 8), the scores of each dimension of risk perception in the vaccinated group were significantly higher than those in the unvaccinated group (all *p* < 0.05), and the total score of risk perception in the vaccinated group was significantly higher (2.72 ± 1.42 vs. 2.26 ± 1.64, *p* < 0.05). The total score of 91.4% for the older adults population was ≤4 points, and they were concentrated in low areas (2–3 points accounted for 52.0%), indicating that the older adults population generally had risk cognition defects.

#### 3.3.2. Attitudes and Behaviors Related to Influenza Vaccine

The cognition of vaccine safety/effectiveness, evaluation of adverse reactions, family vaccination rate, vaccination intention, access to vaccination sites and perceived benefits of vaccination in the vaccinated group were significantly better than those in the unvaccinated group (all *p* < 0.05, Table 3). Specifically, the acceptance rate of vaccine safety in the vaccinated group (70.3%) was significantly higher than that in the unvaccinated group (22.0%). The acceptance rate of vaccine effectiveness was 49.7%, which was significantly higher than that of the unvaccinated group (9.3%). In terms of concerns about influenza vaccine, older adults mainly paid attention to the safety of influenza vaccine (41.2%), followed by the effectiveness (36.1%) and the origin of the vaccine (34.8%), and the price (27.9%) was the lowest.

### 3.4. Influenza- and Influenza Vaccine-Related Knowledge Acquisition and Information Needs

In terms of access to knowledge, a total of seven common ways to obtain information at this stage were included. In the vaccinated group, family members/friends (68.26%), medical institutions (54.56%) and television (51.17%) were the main sources. In the unvaccinated group, the main sources were TV (60.34%), family/friends (51.20%) and medical institutions (46.92%). There were significant differences in the utilization rate of five access ways between the two groups (*p* < 0.05). Both groups expected to acquire knowledge about influenza and influenza vaccine through medical institutions and television in the future. In terms of knowledge content, a total of seven influenza-related knowledge and five influenza vaccine-related knowledge items were set up. Influenza prevention and disease hazards, safety and efficacy of influenza vaccine were the top needs of both groups. The vaccinated group had a significantly higher need for influenza transmission routes than the unvaccinated group (rate difference 15.3%, *p* < 0.05), and the unvaccinated group had a higher need for treatment (rate difference 12.1%, *p* < 0.05). The unvaccinated group had a significantly higher demand for contraindications to influenza vaccination (rate difference 22.4%, *p* < 0.05), and the vaccinated group paid more attention to the time/place of vaccination. (Table 4).

### 3.5. Hypothetical Scenarios Associated with Increased Vaccination Willingness

This section assessed participants’ stated willingness to receive influenza vaccination under 11 hypothetical scenarios, including informing older adults about the safety of influenza vaccine, that influenza vaccination can effectively reduce the chance of influenza infection, that influenza vaccination can reduce the likelihood of infection for other family members, that the cost of influenza vaccination is covered by medical insurance, that influenza vaccination is free of charge, influenza vaccination recommended by a doctor or trusted friend or family member, other older adults receiving influenza vaccination, personal or family experience of safe influenza vaccination, and on-site influenza vaccination offered by health care facilities.

In the vaccinated group, the scenarios most frequently reported as willingness-enhancing were free-of-charge vaccination (endorsed by 66.32%), clear safety information (62.48%), and family protection benefits (62.38%). In the unvaccinated group, free vaccination was also the most commonly cited hypothetical facilitator. Notably, apart from the scenario of ‘others vaccinated demonstration’, over 50% of vaccinated respondents stated they would ‘definitely continue’ to receive the influenza vaccine under most of the proposed conditions, as detailed in Table 5.

### 3.6. Factors Associated with Vaccination Status

A total of 24 socio-demographic information and health awareness variables were included as influencing factors in the univariate analysis. The results showed that some variables, such as age, household registration, and medical insurance type, were significantly associated with vaccination behavior (all *p* < 0.05). Taking whether or not to receive influenza vaccine as the dependent variable, and factors with statistically significant differences in univariate analysis as independent variables, variables related to vaccine cognition and attitudes and beliefs were included to construct a multivariate logistic regression model.

In the fully adjusted model (Model 3), age was positively associated with vaccination (aOR = 1.031, 95%CI: 1.016–1.046). Rural residents had significantly lower vaccination odds than urban residents (aOR = 0.763, 95%CI: 0.627–0.929). Employment status showed a significant inverse association: both part-time (aOR = 0.548, 95%CI: 0.387–0.774) and full-time workers (aOR = 0.413, 95%CI: 0.284–0.602) were less likely to be vaccinated than retirees. The presence of students or children in the family was inversely associated with vaccination (aOR = 0.735, 95%CI: 0.580–0.930). Medical insurance type was significantly associated with vaccination (*p* < 0.001): compared with those without insurance, participants with Resident Basic Medical Insurance (aOR = 0.065, 95%CI: 0.017–0.250), Employee Basic Medical Insurance (aOR = 0.079, 95%CI: 0.020–0.302), and New Rural Cooperative Medical Scheme (aOR = 0.057, 95%CI: 0.015–0.221) all had lower vaccination odds. Hypertension was inversely associated with vaccination (aOR = 0.785, 95%CI: 0.654–0.943).

In terms of knowledge, attitudes, and practices, the fully adjusted model revealed that each 1-point increase in vaccine-related knowledge score was associated with a 24.6% increase in the odds of vaccination (aOR = 1.246, 95%CI: 1.122–1.384). Awareness of influenza vaccine remained the strongest correlate (aOR = 9.151, 95%CI: 6.320–13.251). Perceived vaccine safety (aOR = 1.808, 95%CI: 1.314–2.488) and perceived vaccine effectiveness (aOR = 2.115, 95%CI: 1.453–3.079) were also positively associated with vaccination. Influenza risk perception score was positively associated with vaccination (aOR = 1.088, 95%CI: 1.027–1.153). (Table 6).

## 4. Discussion

Influenza can cause more than 650,000 deaths worldwide every year, and the excess respiratory mortality rate of older adults over 60 years old is 38.5/100,000 in China [13,14]. Vaccination can reduce the risk of morbidity by 40–60% [15], but the vaccination rate of influenza among older adults over 60 years old in China is only 3.8% [11]. Suzhou’s GDP ranks sixth in China, and its elderly population will reach 3.26 million in 2023. As an economically developed area, the influenza vaccine vaccination rate is less than 1% [12]. This low coverage in an affluent setting underscores the need to identify modifiable correlates of vaccination behavior in economically developed regions. The results of multivariate logistic regression analysis of this study showed that influenza vaccination behavior was affected by social structural factors and influenza- and influenza vaccine-related knowledge, attitude and behavior, which was consistent with previous studies [16].

Our data show that rural household registration (aOR = 0.76, 95%CI: 0.63–0.93) and full-time employment (aOR = 0.41, 95%CI: 0.28–0.60) were inversely associated with vaccination. These findings are consistent with observations from the United States, where rural older adults have consistently lower influenza vaccination rates than their urban counterparts. A study using Medicare data [17] found that, although overall vaccination rates changed little from 2019 to 2022, they increased substantially for Black and Hispanic older adults in rural areas while decreasing for some groups of White older adults. Similarly, a national analysis of Medicaid beneficiaries [18] found that rural residents had lower vaccination odds than urban residents, with the disparity widening over time. In our sample, full-time employed older adults exhibited a stronger inverse association, suggesting that workplace-based vaccination programs or extended clinic hours may warrant consideration in Suzhou.

We also observed that the vaccination rate among participants with Resident Basic Medical Insurance was lower than among those without any insurance. This counterintuitive finding may be explained by the low awareness of reimbursement policy among insured individuals—only 34.4% of our sample were aware that influenza vaccination costs could be covered by personal medical insurance accounts. This suggests that passive policy implementation without active communication may yield limited effects.

Our data reveal substantial gaps in both influenza-related knowledge (4.06 vs. 3.16, *p* < 0.001) and vaccine-related knowledge (2.10 vs. 0.67, *p* < 0.001) between vaccinated and unvaccinated groups. Awareness of influenza vaccine was the variable most strongly associated with vaccination status (aOR = 9.15, 95%CI: 6.32–13.25). This strong association aligns with the KAP model, which posits that knowledge is the foundation of behavior change [19]. This result suggests that older adults may be overly concerned about the complexity or uncertainty of influenza disease, rather than simply lacking knowledge about influenza and its vaccine, which may lead to reduced or even negative responses to vaccination [20]. That is, knowledge deficiency may be a core obstacle to low vaccination rates [16]. These findings are consistent with studies from Germany [21], where older adults who lacked knowledge about vaccine safety and efficacy were significantly less likely to be vaccinated. Studies in European settings have similarly shown that perceived vaccine safety and effectiveness are among the variables most strongly associated with influenza vaccination in older adults.

However, due to the cross-sectional nature of our data, we cannot determine the causal direction of this association. While it is plausible that greater knowledge facilitates vaccination uptake, it is equally plausible that vaccinated individuals acquire knowledge through interactions with healthcare professionals at the time of vaccination—or that both knowledge and vaccination are driven by unmeasured factors such as health-seeking propensity. Future longitudinal studies are needed to disentangle these temporal relationships.

In addition to knowledge, our data show that perceived protection (aOR = 1.18, 95%CI: 0.86–1.60) and having a planned vaccination site (aOR = 2.66, 95%CI: 2.01–3.54) were positively associated with vaccination. The vaccinated group also demonstrated higher endorsement of vaccine safety (70.3% vs. 22.0%) and effectiveness (49.7% vs. 9.3%). These attitudinal differences align with the Health Belief Model, which posits that perceived susceptibility, severity, benefits, and barriers jointly predict health behaviors. The vaccinated group showed higher risk perception and more positive attitudes, which further increased confidence in the value of influenza vaccination and improved the acceptance of influenza vaccination under social norms [22]. This result is consistent with the conclusions of numerous global studies [23,24], indicating that individual perceptions of vaccine safety and efficacy directly affect preventive behaviors.

We found a notable divergence in information sources: vaccinated groups relied more heavily on healthcare institutions (54.6%), whereas unvaccinated groups primarily used television (60.3%). This pattern is consistent with findings from other settings. A study in urban China [25] found that strengthening physician recommendations is a key strategy to reduce vaccine hesitancy and improve coverage. A study in rural southern China [26] found that trust in vaccination service providers was positively associated with vaccination uptake. The unvaccinated group’s reliance on television for information suggests that digital health education may be a breakthrough in future communication, and television public service advertisements could be used to introduce and promote influenza vaccine.

In our hypothetical scenario analysis, free vaccination was endorsed as a willingness-enhancing factor by 66.3% of the vaccinated groups and was also the most commonly cited facilitator among the unvaccinated groups. This pattern is consistent with findings from studies in areas with free vaccination policies, such as Beijing and Sichuan [27,28], where vaccination rates are much higher than in areas without such policies. This survey is consistent with previous studies, suggesting that implementation and advocacy of preferential policies for vaccination costs could greatly improve influenza vaccination rates, especially free vaccination policies [29,30]. Our results showed that 66.32% of vaccinated older adults would continue to receive influenza vaccination under the assumption of free vaccination. A free vaccination policy combined with convenient payment and settlement mechanisms is recommended to improve the convenience and coverage of influenza vaccination in older adults.

Based on our findings and supporting evidence from other settings, we suggest the following approaches may be worth considering for future vaccination promotion efforts. The strong association between vaccine awareness and vaccination status (aOR = 9.15) suggests that health education may be a key component of future strategies. In particular, a “core facts first” communication strategy—prioritizing vaccination location and reimbursement policy information before complex medical details—may be effective. The information source disparities we observed suggest that both healthcare institutions (for personalized recommendations) and television (for broad public awareness campaigns) may be effective channels for different subgroups. The inverse association between full-time employment and vaccination (aOR = 0.41) suggests that extending vaccination clinic hours may facilitate access for employed older adults. For the unvaccinated group, a “problem-solution” strategy could be used for targeted health education, such as addressing concerns about how to safely vaccinate older adults with chronic conditions. Finally, the high endorsement of free vaccination in hypothetical scenarios suggests that cost-related policies may be worth exploring. These policy recommendations, however, are based on our interpretation of the data combined with external evidence, and their effectiveness in the local context would require further evaluation through implementation research.

This study has certain limitations. First, the cross-sectional design precludes any causal inference; we cannot determine whether the associations observed reflect knowledge facilitating vaccination, vaccination promoting knowledge-seeking, or confounding by unmeasured health-seeking behaviors. Second, our sampling frames may have introduced selection bias. The unvaccinated group was drawn exclusively from the basic public health service system (which covers older adults who have established health records and received community health check-ups), potentially excluding the most health-disengaged older adults who never utilize primary care services. Consequently, our findings may be just generalizable to those engaged with community health services. Third, the 11 hypothetical scenarios presented to participants reflect stated preferences rather than actual implemented interventions, and responses may be subject to social desirability bias. Fourth, data on vaccination status were collected through self-report and system verification; however, potential misclassification cannot be fully excluded. Fifth, some confounding from unmeasured variables (e.g., cognitive status, health literacy, prior adverse vaccine experiences) cannot be ruled out. Sixth, the study was conducted in Suzhou, an economically developed city in eastern China; the vaccine supply, income levels, educational attainment, and healthcare infrastructure in other Chinese cities may differ considerably. Therefore, our conclusions may have limited generalizability to less developed regions. Finally, the survey was conducted during a single influenza season (2023–2024); vaccination behavior and its correlates may vary across seasons due to changes in circulating strains, vaccine match, public concern, or outbreak context.

## 5. Conclusions

In summary, our data show that influenza vaccine awareness was the strongest correlate of vaccination behavior (aOR = 9.15, 95%CI: 6.32–13.25), followed by having a planned vaccination site (aOR = 2.66, 95%CI: 2.01–3.54) and perceived vaccine effectiveness (aOR = 2.12, 95%CI: 1.45–3.08). Rural household registration, full-time employment, smoking, and hypertension were inversely associated with vaccination. Information source preferences differed markedly between vaccinated and unvaccinated older adults: healthcare institutions for the former and television for the latter. Free vaccination was the most commonly endorsed hypothetical facilitator. These findings suggest that targeted health education delivered through healthcare professionals, mass media campaigns reaching television audiences, extended clinic hours for employed individuals, and policy measures addressing cost barriers may together be necessary to improve vaccination coverage. Future implementation research is needed to evaluate the feasibility and effectiveness of these approaches in the local context.

## Figures and Tables

**Table 1:** Basic characteristics of study participants (N = 4622).

Characteristics	N	Percentage (%)
Sex		
Male	2197	47.53
Female	2425	52.47
Age (years)		
[60,65)	914	19.77
[65,70)	1273	27.54
[70,75)	1098	23.76
[75,80)	800	17.31
[80,-)	537	11.62
Household type		
Urban	1732	37.47
Rural	2890	62.53
Educational level		
Illiterate/Semi-literate	830	17.96
Primary School	1592	34.44
Junior high school/Technical secondary school	1371	29.66
High school/Vocational high school	631	13.65
Junior College	140	3.03
Undergraduate	58	1.25
Medical insurance type		
No insurance	14	0.30
Resident Basic Medical Insurance	1443	31.22
Employee Basic Medical Insurance	2470	53.44
New Rural Cooperative Medical Scheme	686	14.84
Other commercial insurance	9	0.19
The personal annual income (Chinese Yuan, CNY)		
<50,000 yuan	3227	69.82
50,000–100,000 yuan	1112	24.06
110,000–140,000 yuan	223	4.82
150,000–190,000 yuan	37	0.80
200,000–240,000 yuan	10	0.22
≥250,000 yuan	13	0.28
The family annual income (Chinese Yuan, CNY)		
<50,000 yuan	1285	27.80
50,000–100,000 yuan	1369	29.62
110,000–140,000 yuan	664	14.37
150,000–190,000 yuan	557	12.05
200,000–240,000 yuan	374	8.09
≥250,000 yuan	373	8.07
The Body Mass Index		
<18.5	182	3.94
[18.5,23.9]	2387	51.64
[24,27.9]	1671	36.15
≥28	382	8.26
Hypertension		
Yes	2385	51.60
No	2237	48.40
Diabetes		
Yes	1578	34.14
No	3044	65.86

**Table 2:** Quantitative analysis of knowledge awareness of influenza and influenza vaccine among elderly population in Suzhou.

Related Knowledge	Vaccinated Group	Unvaccinated Group	T-Value	*p*
	Scores (Mean ± SD)	Scores (Mean ± SD)		
Influenza-related knowledge	4.06 ± 2.21	3.16 ± 2.33	13.526	<0.001
Influenza is more serious than a “bad cold”	0.69 ± 0.46	0.56 ± 0.50	8.960	<0.001
Influenza can be severe for a full week	0.53 ± 0.50	0.39 ± 0.49	9.351	<0.001
Influenza can cause serious complications	0.57 ± 0.50	0.40 ± 0.49	11.675	<0.001
Influenza can lead to hospitalization	0.57 ± 0.50	0.40 ± 0.49	11.735	<0.001
Influenza can cause death	0.44 ± 0.32	0.32 ± 0.47	8.115	<0.001
The route of influenza infection or transmission	0.48 ± 0.23	0.43 ± 0.23	7.595	<0.001
Measures to prevent influenza	0.56 ± 0.27	0.49 ± 0.27	9.501	<0.001
Influenza epidemic season in Suzhou city	0.21 ± 0.35	0.16 ± 0.31	5.708	<0.001
Influenza vaccine-related knowledge	2.10 ± 1.18	0.67 ± 1.07	42.406	<0.001
The best time to get the influenza vaccine	0.50 ± 0.50	0.15 ± 0.36	26.453	<0.001
Locations for influenza vaccination	0.36 ± 0.19	0.16 ± 0.23	32.221	<0.001
The frequency of influenza vaccination	0.70 ± 0.46	0.16 ± 0.37	42.906	<0.001
Priority for influenza vaccination	0.33 ± 0.25	0.15 ± 0.25	25.136	<0.001
Reimbursement for influenza vaccination	0.20 ± 0.40	0.07 ± 0.25	12.825	<0.001

**Table 3:** Comparison of influenza- and vaccine-related attitudes and practices between vaccinated and unvaccinated groups.

Items	Vaccinated Group (N = 2007) n (%)	Unvaccinated Group (N = 2615) n (%)	χ^2^	*p*
**Influenza-related attitudes and behaviors**				
Q1. Are you worried about getting influenza yourself?			281.869	<0.001
Never worried	418 (20.8)	1013 (38.7)		
Not worried	1338 (66.7)	1231 (47.1)		
A little worried	170 (8.5)	112 (4.3)		
Very worried	81 (4.0)	259 (9.9)		
Q2. Do you think you are not at risk of severe illness from influenza?			70.155	<0.001
Yes/Don‘t know	970 (48.3)	1587 (60.7)		
No	1037 (51.7)	1028 (39.3)		
Q3. Do you think you are more susceptible to influenza than your peers?			185.984	<0.001
No/Don’t know	1367 (68.1)	2222 (85.0)		
Yes	640 (31.9)	393 (15.0)		
Q4. Are you worried about your family members getting influenza?			150.410	<0.001
Never worried	427 (21.3)	896 (34.3)		
Not worried	1380 (68.8)	1387 (53.0)		
A little worried	121 (6.0)	119 (4.6)		
Very worried	79 (3.9)	213 (8.1)		
**Influenza vaccine-related attitudes and behaviors**				
Q1. Do you think the influenza vaccine is safe?			1471.230	<0.001
Don’t know	319 (15.9)	1817 (69.5)		
Unsafe	10 (0.5)	17 (0.7)		
Not very safe	50 (2.5)	139 (5.3)		
Safe	1411 (70.3)	576 (22.0)		
Very safe	217 (10.8)	66 (2.5)		
Q2. Do you think the influenza vaccine is effective?			1542.041	<0.001
Don’t know	297 (14.8)	1838 (70.3)		
Ineffective	35 (1.7)	29 (1.1)		
Sometimes effective	678 (33.8)	504 (19.3)		
Very effective	997 (49.7)	244 (9.3)		
Q3. Do you think there will be adverse reactions after influenza vaccination?			1811.917	<0.001
Don’t know	232 (11.6)	1561 (59.7)		
There are adverse reactions, so I don’t want to vaccinate	40 (2.0)	228 (8.7)		
Adverse reactions are rare, so vaccination is necessary	1709 (85.2)	576 (22.0)		
I don’t want to vaccinate regardless of adverse reactions	26 (1.3)	250 (9.6)		
Q4. In the past year, have any of your family members received influenza vaccine?			934.677	<0.001
Yes	849 (42.3)	135 (5.2)		
No	1158 (57.7)	2480 (94.8)		
Q5. Have you already planned where to get the influenza vaccine this year?			1637.350	<0.001
Don’t know	555 (27.7)	1931 (73.8)		
Agree	1443 (31.2)	186 (7.1)		
Disagree	693 (15.0)	498 (19.0)		
Q6. Are you not interested in receiving the influenza vaccine?			1427.704	<0.001
Don’t know	387 (19.3)	1791 (68.5)		
Agree	241 (12.0)	418 (16.0)		
Disagree	1379 (68.7)	406 (15.5)		
Q7. Are you worried about side effects of the influenza vaccine?			1041.444	<0.001
Don’t know	421 (21.0)	1710 (65.4)		
Agree	902 (44.9)	733 (28.0)		
Disagree	684 (34.1)	172 (6.6)		
Q8. There is no convenient place for you to get vaccinated			1239.596	<0.001
Don’t know	283 (14.1)	1700 (65.0)		
Agree	61 (3.0)	88 (3.4)		
Disagree	1663 (82.9)	827 (31.6)		
Q9. Do you feel that you must get vaccinated?			1504.399	<0.001
Don’t know	482 (24.0)	1780 (68.1)		
Agree	1158 (57.7)	173 (6.6)		
Disagree	367 (18.3)	662 (25.3)		
Q10. Getting the influenza vaccine can protect you from influenza infection			1337.532	<0.001
Don’t know	354 (17.6)	1820 (69.6)		
Agree	1506 (75.0)	593 (22.7)		
Disagree	147 (7.3)	202 (7.7)		
Q11. Getting the influenza vaccine is worth the time and cost			1571.546	<0.001
Don’t know	358 (17.8)	1833 (70.1)		
Agree	1506 (75.0)	457 (17.5)		
Disagree	143 (7.1)	325 (12.4)		

**Table 4:** Sources, preferred channels, and information needs regarding influenza and influenza vaccination among older adults in Suzhou.

Items	Vaccinated Group (N = 2007) n (%)	Unvaccinated Group (N = 2615) n (%)	χ^2^	*p*
**Sources of influenza prevention knowledge (actual channels)**				
Television	1027 (51.17)	1578 (60.34)	38.849	<0.001
Radio	423 (21.08)	614 (23.48)	3.770	0.052
Newspapers/Magazines	366 (18.24)	459 (17.55)	0.362	0.547
Mobile phones	780 (38.86)	994 (38.01)	0.349	0.555
Billboards/Manuals	162 (8.07)	210 (8.03)	0.003	0.959
Family/Friends	1370 (68.26)	1339 (51.20)	136.177	<0.001
Medical institutions	1095 (54.56)	1227 (46.92)	26.494	<0.001
Disease Control and Prevention Agencies	200 (9.97)	211 (8.07)	5.040	0.025
**Preferred channels for acquiring influenza prevention knowledge**				
Television	1240 (61.78)	1776 (67.92)	18.832	<0.001
Radio	623 (31.04)	868 (33.19)	2.406	0.121
Newspapers/Magazines	563 (28.05)	638 (24.40)	7.883	0.005
Mobile phones	919 (45.79)	1126 (43.06)	3.432	0.064
Billboards/Manuals	375 (18.68)	408 (15.60)	7.667	0.006
Family/Friends	1160 (57.80)	1287 (49.22)	33.566	<0.001
Medical institutions	1335 (66.52)	1529 (58.47)	31.197	<0.001
Disease Control and Prevention Agencies	480 (23.92)	512 (19.58)	12.671	<0.001
**Influenza-related knowledge and information needs**				
Epidemiological characteristics	907 (45.19)	1067 (40.80)	8.939	0.003
Transmission routes	1237 (61.63)	1473 (56.33)	13.177	<0.001
Disease severity	1246 (62.08)	1584 (60.57)	1.089	0.297
Prevention measures	1627 (81.07)	1923 (73.54)	36.133	<0.001
Treatment methods	1210 (60.29)	1558 (59.58)	0.238	0.626
Epidemic trends	560 (27.90)	612 (23.40)	12.143	<0.001
Influenza vaccine-related information	621 (30.94)	541 (20.69)	63.431	<0.001
**Influenza vaccine-related knowledge and information needs**				
Requirements and contraindications for vaccination	1362 (67.86)	1587 (60.69)	25.306	<0.001
Vaccination time and location	1388 (69.16)	1420 (54.30)	105.102	<0.001
Safety	1689 (84.16)	2142 (81.91)	4.029	0.049
Vaccine effectiveness	1439 (71.70)	1684 (64.40)	27.624	<0.001
Vaccination strategy	568 (28.30)	555 (21.22)	30.921	<0.001

**Table 5:** Proportion of respondents stating willingness to receive influenza vaccination under 11 hypothetical scenarios, by prior vaccination status.

Hypothetical Facilitators	Vaccinated Group (N = 2007)	Unvaccinated Group (N = 2615)	χ^2^	*p*
	n (%)	n (%)		
Influenza vaccine is safe	1254 (62.48)	336 (12.85)	1239.517	<0.001
Influenza vaccination can effectively reduce the chance of influenza infection	1237 (61.63)	316 (12.08)	1249.599	<0.001
Influenza vaccination reduces infection in family members	1252 (62.38)	367 (14.03)	1166.246	<0.001
Influenza vaccination is covered by health insurance	1208 (60.19)	382 (14.61)	1045.433	<0.001
The influenza vaccine is free	1331 (66.32)	555 (21.22)	955.943	<0.001
Recommended by a doctor	1087 (54.16)	338 (12.93)	905.365	<0.001
Recommended by a trusted friend or family member	1090 (54.31)	330 (12.62)	927.282	<0.001
Other older adults were vaccinated	896 (44.64)	237 (9.06)	776.866	<0.001
Experience in safe influenza vaccination	1106 (55.11)	321 (12.28)	976.083	<0.001
Family members had experience of safe influenza vaccination	1139 (56.75)	369 (14.11)	939.232	<0.001
On-site vaccination is provided by health care facilities	1027 (51.17)	311 (11.89)	851.703	<0.001

**Table 6:** Factors associated with influenza vaccination among older adults in Suzhou.

Variable	Model 1 aOR (95% CI)	*p*	Model 2 aOR (95% CI)	*p*	Model 3 aOR (95% CI)	*p*
(a) Sociodemographic and health-related characteristics						
Age	1.021 (1.011–1.031)	<0.001	1.036 (1.023–1.049)	<0.001	1.031 (1.016–1.046)	<0.001
Gender (ref: female)	0.822 (0.713–0.948)	0.007	0.783 (0.658–0.931)	0.006	0.780 (0.639–0.953)	0.015
Household type (ref: rural)	1.360 (1.182–1.565)	<0.001	1.313 (1.107–1.557)	0.002	1.310 (1.075–1.595)	0.007
Education (ref: illiterate/semi-literate)		<0.001		0.034		0.059
Primary school	1.708 (1.413–2.066)	<0.001	1.368 (1.081–1.731)	0.009	1.364 (1.044–1.782)	0.023
Middle school	1.882 (1.527–2.321)	<0.001	1.291 (0.996–1.672)	0.053	1.201 (0.894–1.614)	0.225
High school	1.618 (1.256–2.084)	<0.001	1.009 (0.740–1.375)	0.955	0.936 (0.657–1.332)	0.713
College	3.064 (1.965–4.778)	<0.001	1.443 (0.852–2.445)	0.173	1.445 (0.786–2.654)	0.236
Bachelor or above	2.343 (1.281–4.288)	0.006	1.238 (0.597–2.567)	0.566	1.294 (0.550–3.046)	0.555
Medical insurance type (ref: no insurance)		<0.001		<0.001		<0.001
Resident Basic Medical Insurance	0.259 (0.078–0.863)	0.028	0.06 (0.016–0.217)	<0.001	0.065 (0.017–0.250)	<0.001
Employee Basic Medical Insurance	0.282 (0.085–0.940)	0.039	0.065 (0.018–0.236)	<0.001	0.079 (0.020–0.302)	<0.001
New Rural Cooperative Medical Scheme (NRCMS)	0.184 (0.055–0.619)	0.006	0.051 (0.014–0.186)	<0.001	0.057 (0.015–0.221)	<0.001
Other commercial insurance	0.392 (0.062–2.461)	0.318	0.225 (0.030–1.674)	0.145	0.208 (0.021–2.039)	0.178
Current Employment status (ref: retired)		<0.001		<0.001		<0.001
Current part-time job	0.625 (0.487–0.801)	<0.001	0.537 (0.399–0.721)	<0.001	0.548 (0.387–0.774)	0.001
Current full-time job	0.453 (0.345–0.595)	<0.001	0.396 (0.286–0.550)	<0.001	0.413 (0.284–0.602)	<0.001
Whether there are students or children in the family (ref: no)	0.750 (0.632–0.890)	0.001	0.774 (0.630–0.951)	0.015	0.735 (0.580–0.930)	0.010
Personal annual income (ref: <50,000 yuan)		0.010		0.037		0.076
50,000–100,000 yuan	1.255 (1.064–1.479)	0.007	1.296 (1.061–1.582)	0.011	1.302 (1.033–1.642)	0.026
110,000–140,000 yuan	1.673 (1.200–2.331)	0.002	1.674 (1.113–2.518)	0.013	1.802 (1.135–2.860)	0.013
150,000–190,000 yuan	1.925 (0.905–4.098)	0.089	2.094 (0.880–4.985)	0.095	2.036 (0.792–5.230)	0.140
200,000–240,000 yuan	0.714 (0.170–3.009)	0.647	0.647 (0.118–3.532)	0.615	0.930 (0.124–6.950)	0.944
≥250,000 yuan	1.635 (0.511–5.226)	0.407	1.577 (0.390–6.376)	0.523	1.856 (0.401–8.601)	0.429
Smoking (ref: no)	0.530 (0.432–0.650)	<0.001	0.523 (0.409–0.669)	<0.001	0.604 (0.453–0.805)	0.001
Self-rated health status (ref: poor)		<0.001		0.005		0.015
Fair	0.992 (0.711–1.386)	0.963	0.988 (0.657–1.486)	0.954	1.139 (0.705–1.841)	0.595
Good	1.330 (0.950–1.863)	0.096	1.232 (0.815–1.860)	0.322	1.517 (0.933–2.468)	0.093
Very good	1.633 (1.152–2.315)	0.006	1.465 (0.956–2.246)	0.079	1.642 (0.991–2.720)	0.054
Excellent	1.414 (0.912–2.193)	0.122	1.358 (0.794–2.323)	0.264	1.408 (0.748–2.649)	0.289
Diabetes (ref: no)	0.888 (0.775–1.017)	0.087	0.940 (0.796–1.109)	0.462	1.004 (0.829–1.216)	0.968
Hypertension (ref: no)	0.941 (0.827–1.071)	0.359	0.848 (0.724–0.993)	0.041	0.785 (0.654–0.943)	0.009
Cancer (ref: no)	1.389 (1.039–1.857)	0.026	1.368 (0.957–1.957)	0.086	1.589 (1.055–2.393)	0.027
(b) Knowledge-related variables						
Influenza-related knowledge score			0.872 (0.837–0.908)	<0.001	0.823 (0.785–0.864)	<0.001
Vaccine-related knowledge score			1.924 (1.770–2.091)	<0.001	1.246 (1.122–1.384)	<0.001
Awareness of influenza (ref: no)			1.366 (1.033–1.806)	0.029	1.130 (0.827–1.546)	0.443
Level of influenza knowledge (ref: nothing)				0.114		0.058
A little			0.881 (0.673–1.153)	0.355	0.921 (0.682–1.243)	0.590
Some			0.872 (0.639–1.188)	0.385	0.893 (0.630–1.265)	0.523
A lot			0.478 (0.272–0.842)	0.011	0.420 (0.221–0.802)	0.009
A great deal			0.337 (0.065–1.748)	0.195	0.201 (0.035–1.159)	0.073
Awareness of influenza vaccine (ref: no)			7.938 (5.544–11.365)	<0.001	9.151 (6.320–13.251)	<0.001
Level of vaccine knowledge (ref: nothing)				0.064		0.438
A little			1.163 (0.856–1.580)	0.334	0.678 (0.425–1.083)	0.104
Some			1.513 (1.051–2.180)	0.026	0.725 (0.428–1.229)	0.232
A lot			1.904 (0.970–3.737)	0.061	0.776 (0.329–1.827)	0.562
A great deal			4.362 (0.536–35.467)	0.168	2.186 (0.202–23.665)	0.520
(c) Health attitudes and behaviors						
Vaccine safety (ref: don’t know)						<0.001
Unsafe					2.035 (0.726–5.703)	0.176
Not so safe					0.918 (0.568–1.486)	0.729
Safe					1.808 (1.314–2.488)	<0.001
Very safe					1.483 (0.915–2.405)	0.110
Vaccine effectiveness (ref: don’t know)						<0.001
Not effective					6.273 (3.175–12.396)	<0.001
Sometimes effective					2.011 (1.441–2.805)	<0.001
Very effective					2.115 (1.453–3.079)	<0.001
Influenza risk perception score					1.088 (1.027–1.153)	0.004
Behavioral readiness (ref: don’t know)						<0.001
No planned vaccination					0.458 (0.347–0.603)	<0.001
Schedule of vaccination					2.664 (2.007–3.536)	<0.001
Vaccination safety concerns (ref: don’t know)						<0.001
Do not worry about side effects					1.147 (0.797–1.650)	0.460
Worried about side effects of vaccination					0.609 (0.448–0.830)	0.002
Accessibility to services (ref: don’t know)						<0.001
Convenient location for vaccination					1.748 (1.201–2.544)	0.004
Inconvenient vaccination sites					0.712 (0.407–1.246)	0.234
Subjective norms (ref: don’t know)						<0.001
Non-mandatory vaccination					0.492 (0.370–0.655)	<0.001
Vaccination is mandatory					1.852 (1.335–2.569)	<0.001
Perceived protection (ref: don’t know)						0.290
No perceived protection from vaccination					0.933 (0.627–1.387)	0.730
Perceived vaccination protection					1.175 (0.861–1.603)	0.310

Note: Model 1 included sociodemographic and health-related characteristics only. Model 2 additionally included knowledge variables. Model 3 further included health attitudes and behaviors. All models were adjusted for age, gender, education, medical insurance, employment status, income, smoking, self-rated health, and chronic conditions. Values are presented as adjusted odds ratios (aOR) with 95% confidence intervals (CI).

## Data Availability

Data will be made available on request.

## Supplementary Materials

The following supporting information can be downloaded at: https://www.mdpi.com/article/10.3390/vaccines14070645/s1, Supplementary S1: Questionnaire.
