# Supplementary material for: Influenza Vaccination Status and Associated Factors Among Older Adults in Suzhou, China: A Comparative Cross-Sectional Survey
Source: Vaccines (Basel). 2026 Jul 22;14(7):645. doi: 10.3390/vaccines14070645 (PMC13417161; doi:10.3390/vaccines14070645)
Supplement: Supplementary file 1 [file vaccines-14-00645-s001.zip › vaccines-4412630-supplementary.pdf]

Supplementary S1: Questionnaire

Survey on Flu Knowledge, Attitudes and Behaviors of Older Adults

Survey Form Number:□□□□□

**Ⅰ.Basic Information**	
**A1Name:**	**A2 Gender: ①Male②Female**
**A3ID Number:**	**A4Age: years old**
**A5Current Address: City District/Municipality Street/Village Community/Village Building/Unit/Door Number**	
**A6Your household registration nature belongs to:**	
**①Urban**	**②Rural**
**A7How long have you been living in Suzhou?**	
**①Less than 6 months**	**②6 months or more**
**A8Your educational background:**	
**①Illiterate or semi-literate ②Primary school ③Junior high school / technical secondary school ④High school / vocational high school ⑤College ⑥Bachelor's degree ⑦Graduate or above**	
**A9What jobs have you held before the age of 60?**	
**①Professional technical personnel (healthcare professionals, teachers, laboratory technicians, computer technicians, lawyers, government workers, clerical workers)**<br>**②Management positions (managers, principals)**<br>**③Sales and service workers (retail salespeople, chefs, shopkeepers, hairdressers)**<br>**④Construction, machinery, production, transportation and related workers (factory workers, truck drivers)**<br>**⑤Agricultural, forestry, animal husbandry and fishery workers (farmers, hunters, aquaculture workers, forestry workers)**<br>**⑥Military, police, security (armed forces, police, security guards)**<br>**⑦ Other skilled manual labor (blacksmiths, butchers, tailors or handicraft manufacturers)**<br>**⑧Other unskilled manual labor (miners, cleaners, street vendors, garbage collectors or wood/ water collectors)**<br>**⑨Housewives (full-time housewives, caregivers)**<br>**⑩Unemployed**<br>⑪Others, please specify:___________________	
**A10 Which of the following types does your medical insurance belong to?**	

① Resident Medical Insurance ② Employee Medical Insurance ③ New Rural Cooperative Medical Care ④ Other Commercial Insurance ⑤ No Insurance				
**Ⅱ.Life History in Old Age**				
**B1Do you have a full-time or part-time job now?**				
①No, retired (jump to B3)	②Yes, part-time job	③Yes, full-time job		
**B1.1What type of job do you currently have?**				
①Professional technicians (healthcare professionals, teachers, laboratory technicians, computer technicians, lawyers, government workers, clerical workers)<br>②Management positions (managers, principals)<br>③Sales and service workers (retail salespeople, chefs, shopkeepers, hairdressers)<br>④Construction, machinery, production, transportation and related workers (factory workers, truck drivers)<br>⑤Agriculture, forestry, animal husbandry and fishery workers (farmers, hunters, aquaculture workers, forestry workers)<br>⑥Military, police, security (armed forces, police, security guards)<br>⑦ Other skilled manual labor (blacksmiths, butchers, tailors or handicraft manufacturers)<br>⑧Other unskilled manual labor (miners, cleaners, street vendors, garbage collectors or wood/ water collectors)<br>⑨Housewives (full-time housewives, caregivers)<br>⑩Unemployed<br>⑪Other, please specify:________________				
**B1.2How many hours do you work per week currently?_________Hours**				
**B2Besides you, how many people live with you at home?**<br>(Co-residents refer to all the people living in the same house as you, excluding you yourself.)				
**B2.1What is the relationship between the co-residents and you? (Multiple options are available)**				
①Husband/ Wife	②Father/Mother/In-law	③Son/Stepson/daughter/Stepdaughter	④Grandson/Granddaughter/Grandson-in-law/Granddaughter-in-law	⑤Others, please specify

B3How many children do you have?

Ⅲ.Social and economic status

C1Your personal annual income last year (yuan)?					
①<50,000 yuan	②50,000-100,000 yuan	③110,000-140,000 yuan	④150,000-190,000 yuan	⑤200,000-240,000 yuan	⑥≥250,000 yuan

C2The total income of your family members living with you last year (yuan)?					
①<50,000 yuan	②50,000-100,000 yuan	③110,000-140,000 yuan	④150,000-190,000 yuan	⑤200,000-240,000 yuan	⑥≥250,000 yuan

C3Do you and your family members who live together own or rent the current house or apartment you are in?		
①Own	②Rent	③Don't know

C4Did you need to see a doctor or receive medical care for a period of time last year, but couldn't because of the cost?		
①Yes	②No	③Don't know

C5How difficult is it for you to have enough money to pay for your house, food and other monthly bills?				
①Not at all difficult	②Not very difficult	③A little difficult	④Very difficult	⑤Extremely difficult

Ⅳ. Health and Lifestyle	
D1What is your weight? kilograms	D2 What is your height?__________cm

D3 Are you currently a smoker, such as a cigarette or cigar?	①Yes	②No(Jump to D4)

D3.1 How many years have you been smoking?	____ years
D3.2How many cigarettes do you smoke per day?	Daily____ cigarettes

D4Have you ever smoked?	①Yes	②No(Jump to D5)
D4.1When did you quit smoking?	____ years	

D4.2How many years have you been smoking?	____ years			
D4.3How many cigarettes do you usually smoke per day?	Daily____ cigarettes			
D5Recall over the past month, how was your overall health condition?				
①Very good	②Good	③Fair	④Average	⑤Poor
D6Have you ever been diagnosed by a doctor with heart problems, such as heart attack, congestive heart failure, or angina pectoris?				
①Yes	②No	③Don't know		
D7Have you ever been diagnosed with high blood pressure by a doctor?				
①Yes	②No	③Don't know		
D8Have you ever been diagnosed with a stroke by a doctor?				
①Yes	②No	③Don't know		
D9Have you ever been diagnosed by a doctor with asthma, chronic obstructive pulmonary disease (COPD), emphysema, chronic bronchitis, or any other chronic lung disease?				
①Yes	②No	③Don't know		
D10Have you been diagnosed by a doctor with any kidney diseases?				
①Yes	②No	③Don't know		
D11 Have you been diagnosed by a doctor with liver diseases, including chronic liver diseases?				
①Yes	②No	③Don't know		
D12Have you been diagnosed with a tumor (cancer) by a doctor? (Multiple choices are allowed)				
① No ② Breast ③ Cervical ④ Colon and Rectum ⑤ Liver ⑥ Lung ⑦ Skin ⑧ Prostate ⑨ Others______________				
D13Have you been diagnosed with depression or anxiety disorder by a doctor?				
①Yes	②No	③Don't know		
D14Have you been diagnosed with diabetes by a doctor?				
①Yes	②No	③Don't know		
D15Have you ever been diagnosed by a doctor with any neurological disorders, such as epilepsy or any kind of seizure, or Parkinson's disease?				
①Yes	②No	③Don't know		
D16Have you ever been diagnosed with osteoarthritis by a doctor?				

<table>
<tr><td colspan="4">①Yes</td><td colspan="4">②No</td><td colspan="4">③Don't know</td></tr>
<tr><td colspan="12">D17Have you been diagnosed by a doctor with any autoimmune diseases, such as lupus or rheumatoid arthritis?</td></tr>
<tr><td colspan="4">①Yes</td><td colspan="4">②No</td><td colspan="4">③Don't know</td></tr>
<tr><td colspan="12">D18Have you been diagnosed by a doctor with any other health problems or conditions? (Multiple choices are allowed)</td></tr>
<tr><td colspan="12">① None ② Digestive system diseases ③ Hypothyroidism ④ Skin problems such as eczema ⑤ Other problems or conditions</td></tr>
<tr><td colspan="12">Ⅴ. Influenza Knowledge</td></tr>
<tr><td colspan="12">E1Have you heard of the influenza virus?</td></tr>
<tr><td colspan="6">①Yes</td><td colspan="6">②No</td></tr>
<tr><td colspan="12">E2How much do you know about the influenza virus and the diseases it causes?</td></tr>
<tr><td colspan="3">①Nothing</td><td colspan="2">②A little</td><td colspan="3">③Some</td><td colspan="2">④A lot</td><td colspan="2">⑤A lot of</td></tr>
<tr><td colspan="12">E3Your understanding of the serious adverse consequences of influenza</td></tr>
<tr><td colspan="6">E3.1Influenza is more serious than a "bad cold"</td><td colspan="2">①Yes</td><td colspan="2">②No</td><td colspan="2">③Don't know</td></tr>
<tr><td colspan="6">E3.2 Influenza can be severe for a full week</td><td colspan="2">①Yes</td><td colspan="2">②No</td><td colspan="2">③Don't know</td></tr>
<tr><td colspan="6">E3.3 Influenza can cause serious complications</td><td colspan="2">①Yes</td><td colspan="2">②No</td><td colspan="2">③Don't know</td></tr>
<tr><td colspan="6">E3.4 Influenza can lead to hospitalization</td><td colspan="2">①Yes</td><td colspan="2">②No</td><td colspan="2">③Don't know</td></tr>
<tr><td colspan="6">E3.5 Influenza can cause death</td><td colspan="2">①Yes</td><td colspan="2">②No</td><td colspan="2">③Don't know</td></tr>
<tr><td colspan="12">E4 Do you know the ways of infection or transmission of influenza? (Multiple choices are allowed)</td></tr>
<tr><td colspan="12">① Coughing, sneezing and close conversation ② In an unventilated environment (such as senior activity rooms, chess rooms, etc.)③ Handshaking, hugging ④ Direct contact with hands (such as door handles) ⑤ Food ⑥ Other ________</td></tr>
<tr><td colspan="12">E5 Do you know which preventive measures for influenza? (Multiple choices are allowed)</td></tr>
<tr><td colspan="12">① Get the influenza vaccine ② Wash hands frequently with water and soap ③ Avoid crowds ④ Wear a mask when going to crowded places ⑤ Avoid close contact with influenza patients (or those with cough symptoms) ⑥ Ventilate indoor air and</td></tr>
</table>

keep the room clean⑦ Take some medications for prevention ⑧ Others__________					
E6 Do you think the influenza epidemic season in Suzhou area is? (Multiple choices are allowed)					
①All year round	②Spring	③Summer	④Autumn	⑤Winter	⑥Don't know
**Ⅵ.Attitudes and Behaviors towards Influenza**					
F1Are you worried that you might contract influenza?					
①Not worried	②A little worried	③Very worried	④Never worried		
F2Are you worried that your family members will contract influenza?					
①Yes	②No	③Don't know			
F3Are you worried that your family members will contract influenza?					
①Yes	②No	③Don't know			
F4Are you worried that your family members will contract influenza?					
①Not worried	②A little worried	③Very worried	④Never worried		
**VIII. Knowledge about Influenza Vaccine**					
G1Have you heard of the influenza vaccine?					
①Yes	②No（Jump to I1）				
G2How much do you know about the influenza vaccine?					
①Nothing (Jump to I1)	②A little	③Some	④A lot	⑤A lot of	
G3Do you think this is the best time to get the influenza vaccine?					
①March-May	②June-August	③September-November	④December-February	⑤Other time	⑥Don't know
G4Do you know where to get the influenza vaccine? (Multiple choices are allowed)					
①General hospital	②Disease prevention and control center	③Community health service center (township health center)	④Rural doctor	⑤Don't know	
G5Do you know the frequency of influenza vaccine administration?					
① Lifelong, only one dose ② Every 5 years or more ③ Every 2-5 years ④ Every year ⑤ Don't know					
G6Do you know which groups of people should be prioritized for the influenza					

vaccine? (Multiple choices are allowed)

① Children under 5 years old ② People over 60 years old ③ Patients with chronic diseases ④ Pregnant women ⑤ Medical workers ⑥ Don't know ⑦ Others___________

G7If you have insurance, can the cost of the influenza vaccine be reimbursed?

①Yes, can be reimbursed in full	②Yes, can be reimbursed in full	③No	④Don't know

VIII. Attitudes and Behaviors towards Influenza Vaccine

H1Do you think the influenza vaccine is safe?

①Very safe	②Safe	③Not so safe	④Unsafe	⑤Don't know

H2 Do you think there will be adverse reactions after getting the influenza vaccine?

① There will be adverse reactions, so I don't want to get vaccinated ② Adverse reactions are rare, so it is necessary to get vaccinated ③ No matter whether there are adverse reactions or not, I don't want to get vaccinated ④ Others

H3 Do you think the influenza vaccine is effective?

①Very effective	②Sometimes effective	③Ineffective	④Don't know

H4 What do you consider first when choosing the influenza vaccine?

① Price ② Safety ③ Effectiveness ④ Origin of the vaccine (imported vaccines preferred)

Please rank all the options in the following order: \ \ \

H5 In the past year, did your family get the influenza vaccine?

①Yes	②No	③Unclear

H6In the past year, have you received the influenza vaccine? (The investigator will further verify based on the vaccination record)

①Yes	②No

H7 Regarding the statement about vaccination preparations, do you agree?

H7.1 You have planned where to get the influenza vaccine this year

**①Agree**	**②Disagree**	**③Don't know**
**H7.2 You are not interested in getting the influenza vaccine**		
**①Agree**	**②Disagree**	**③Don't know**
**H7.3 You think you don't need to get the influenza vaccine**		
**①Agree**	**②Disagree**	**③Don't know**
**H8 Regarding the statement about the safety of the influenza vaccine, do you agree?**		
**H8.1 You are worried about the side effects of the influenza vaccine**		
**①Agree**	**②Disagree**	**③Don't know**
**H8.2 Getting the influenza vaccine is safe**		
**①Agree**	**②Disagree**	**③Don't know**
**H8.3 The influenza vaccine may cause you to get the flu**		
**①Agree**	**②Disagree**	**③Don't know**
**H8.4 Severe adverse reactions of the influenza vaccine are very rare**		
**①Agree**	**②Disagree**	**③Don't know**
**H8.5 The influenza vaccine may make you feel nauseous**		
**①Agree**	**②Disagree**	**③Don't know**
**H9 Regarding the statement about the inconvenience of obtaining the influenza vaccine, do you agree?**		
**H9.1 You usually don't have time to get the flu vaccine.**		
**①Agree**	**②Disagree**	**③Don't know**
**H9.2 There is no convenient place for you to get the vaccine.**		
**①Agree**	**②Disagree**	**③Don't know**
**H9.3 Due to inconvenient transportation, it's not convenient for you to get the flu vaccine.**		
**①Agree**	**②Disagree**	**③Don't know**
**H9.4 It's difficult for you to find time to get the flu vaccine.**		
**①Agree**	**②Disagree**	**③Don't know**
**H9.5 You know where to get the flu vaccine.**		
**①Agree**	**②Disagree**	**③Don't know**
**H10 The following statements about your attitude towards getting vaccinated, do you agree?**		
**H10.1 You think you must get vaccinated.**		
**①Agree**	**②Disagree**	**③Don't know**
**H10.2 If you don't get the flu vaccine and eventually get the flu, you will be angry that**		

**you didn't get vaccinated.**		
**①Agree**	**②Disagree**	**③Don't know**
**H10.3 If you don't get the flu vaccine, you will regret it.**		
**①Agree**	**②Disagree**	**③Don't know**
**H10.4 If you don't get the flu vaccine, you will think you will get the flu this year.**		
**①Agree**	**②Disagree**	**③Don't know**
**H10.5 If you get the flu vaccine, you won't be so worried about getting the flu.**		
**①Agree**	**②Disagree**	**③Don't know**
**H10.6 If you don't get the flu vaccine, you will think it's easy to get the flu.**		
**①Agree**	**②Disagree**	**③Don't know**
**H10.7 You are healthy and there is no need for you to get the flu vaccine.**		
**①Agree**	**②Disagree**	**③Don't know**
**H11 The following statements about the benefits of getting vaccinated, do you agree?**		
**H11.1 Getting the flu vaccine can protect you from getting the flu.**		
**①Agree**	**②Disagree**	**③Don't know**
**H11.2 Getting the flu vaccine is worth spending time and money.**		
**①Agree**	**②Disagree**	**③Don't know**
**H11.3 The flu vaccine is not always effective.**		
**①Agree**	**②Disagree**	**③Don't know**
**H11.4 Even if you get the vaccine, you may still get the flu.**		
**①Agree**	**②Disagree**	**③Don't know**
**H12 The following statements about the social impact of getting vaccinated, do you agree?**		
**H12.1 Your doctor suggests that you get the flu vaccine once a year.**		
**①Agree**	**②Disagree**	**③Don't know**
**H12.2 A family member or friend discourages you from getting the flu vaccine.**		
**①Agree**	**②Disagree**	**③Don't know**
**H12.3 Hearing that others get the flu vaccine will encourage you to get the flu vaccine too.**		
**①Agree**	**②Disagree**	**③Don't know**
**IX. Methods for Obtaining Knowledge on Influenza Prevention and Control**		
**I1How do you acquire knowledge about preventing influenza? (Multiple choices are allowed)**		

① Television ② Radio ③ Newspapers/Magazines ④ Mobile phones ⑤ Billboards/Manuals ⑥ Family/Friends ⑦ Medical institutions⑧ Disease Control and Prevention Agencies ⑨ Others________

I2Which methods do you prefer to use to obtain knowledge about preventing influenza? (Multiple choices are allowed)

① Television ② Radio ③ Newspapers/Magazines ④ Mobile phones ⑤ Billboards/Manuals ⑥ Family/Friends ⑦ Medical institutions⑧ Disease Control and Prevention Agencies ⑨ Others________

I3What knowledge and information related to influenza do you need? (Multiple choices are allowed)

① Epidemiological characteristics ② Transmission routes ③ Disease severity ④ Prevention measures ⑤ Treatment methods ⑥ Epidemic trends ⑦ Influenza vaccine-related information ⑧ Others_____________

I4Which knowledge and information related to influenza vaccines do you hope to obtain? (Multiple choices are allowed)

① Requirements and contraindications for vaccination ② Vaccination time and location ③ Safety ④ Vaccine effectiveness ⑤ Vaccination strategy ⑥ Others _____________

X. Evaluation of Possible Interventions

J1Will the following factors affect whether you get the influza vaccine?

	Not get vaccinated	Probably get vaccinated	Definitely get vaccinated
J1.1The influenza vaccine is safe.			
J1.2The influenza vaccine can effectively reduce the chance of getting the flu in the next quarter.			
J1.3The influenza vaccine can reduce the possibility of other family members getting infected.			
J1.4Influenza vaccination is covered by medical insurance.			

**J1.5The influenza vaccine is free of charge.**			
**J1.6Doctors recommend it.**			
**J1.7Trusted friends or family members recommend it.**			
**J1.8Other elderly people have received the influenza vaccine safely.**			
**J1.9There is experience of safe influenza vaccination at the medical institution.**			
**J1.10There is experience of safe influenza vaccination among family members.**			
**J1.11The medical institution provides on-site vaccination.**			

**Your contact information:**	
	**Investigator:**
	**Survey Date: Year Month Day**
